# Effects of Long-Term Exposure to Traffic-Related Air Pollution on Lung Function in Children

**DOI:** 10.1007/s11882-017-0709-y

**Published:** 2017-05-27

**Authors:** Erica S. Schultz, Augusto A. Litonjua, Erik Melén

**Affiliations:** 1 Institute of Environmental Medicine, Karolinska Institute, Box 210, SE-171 77 Stockholm, Sweden; 2 Channing Division of Network Medicine, Department of Medicine, Brigham and Women’s Hospital and Harvard Medical School, 181 Longwood Ave, Boston, MA USA; 3 Sachs’ Children’s Hospital, Södersjukhuset, Stockholm, Sweden; 4 Centre for Occupational and Environmental Medicine, Stockholm County Council, Stockholm, Sweden

**Keywords:** Adolescence, Asthma, Cohort, Epidemiology, Sensitization, Small airways, Spirometry

## Abstract

Lung function in early life has been shown to be an important predictor for peak lung function in adults and later decline. Reduced lung function per se is associated with increased morbidity and mortality. With this review, we aim to summarize the current epidemiological evidence on the effect of traffic-related air pollution on lung function in children and adolescents. We focus in particular on time windows of exposure, small airway involvement, and vulnerable sub-groups in the population. Findings from studies published to date support the notion that exposure over the entire childhood age range seems to be of importance for lung function development. We could not find any conclusive data to support evidence of sup-group effects considering gender, sensitization status, and asthma status, although a possibly stronger effect may be present for children with asthma. The long-term effects into adulthood of exposure to air pollution during childhood remains unknown, but current studies suggest that these deficits may be propagated into later life. In addition, further research on the effect of exposure on small airway function is warranted.

## Introduction

Air pollution (outdoor and indoor) is a global problem and one of the most important environmental determinants for human health. About 300 million children worldwide breathe highly toxic air, defined as levels six or more times exceeding international guidelines [1]. Indoor and outdoor air pollution is linked to 1 in 10 deaths in children under 5 years of age, and out of these, about 20% are attributable to outdoor air pollution levels [2]. The most important process contributing to levels of ambient air pollution in urban settings relates to the combustion of fuels. Due to the proximity between people and sources, road traffic is particularly important for the population exposure to ambient air pollution in developed countries.

Lung development starts in utero, and exposure to air pollution prenatally has been shown to negatively affect respiratory health [3]. Considerable maturation of the lungs continues after birth, which makes the lungs potentially vulnerable to the effects from exposure to air pollution also postnatally. Infants are relatively immobile and are often in a pram during outdoor transportation, at the level of motor exhaust emissions. Infants and children may also be more exposed to air pollution compared to adults relative to their size, due to higher ventilation per minute. In addition, the immune system of infants and young children is not fully developed, which may contribute to an increased vulnerability to the effects of exposure to air pollution [4].

Lung function in early life has been shown to be an important predictor for peak lung function in adults and later decline [5, 6]. Reduced lung function per se is associated with increased morbidity and mortality, even among healthy non-smoking individuals with only modestly reduced lung function [7, 8]. Longitudinal studies have revealed that, taking height and gender into consideration, the deviation of an individual’s value of lung function from the population average remains rather constant independent of age. This is known as tracking of lung function, and a person is said to follow his or her own trajectory if crude lung function values are increasing as expected, relative to age, height, and gender [5, 9]. From a public health perspective, it is therefore of great interest to evaluate the effect of modifiable environmental factors such as pre- and postnatal air pollution exposure on children’s lung function, given the potential long-term effects.

With this review, we aim to summarize the current epidemiological evidence on the effect of traffic-related air pollution on lung function in children and adolescents. We will particularly focus on time windows of exposure, small airway involvement, and vulnerable sub-groups in the population.

**Table:** 

Search strategy and selection criteria
We searched the PubMed databases for publications from Jan 2006 to March 2017, with the search terms (TRAP OR (traffic AND(pollut* OR emission* OR PM OR NO OR particle*))) AND (“Lung function” OR spirometry OR “pulmonary function” OR “forced expiratory” OR “FEV*” OR “Impulse oscillometry” OR “forced oscillat* techniq*” OR “FOT” OR “lung volume” OR “small airway*” OR “peripheral airway”) AND (Children* OR “school-age*” OR “pre-school*” OR infant* OR adolesce*),as well as (TRAP OR traffic OR pollut* OR emission* OR PM OR NO OR particle*))) AND (“Impulse oscillometry” OR “small airway*” OR “peripheral airway*”).
From PubMed search, 258 articles were identified. We also identified references from the bibliographies of these publications and from a review article by Gotschi et al. from 2008 [10].
Included articles had longitudinal air pollution data (about 1-year estimates) or proxies for long-term traffic-related air pollution (like traffic counts/density). In summary, 32 articles with cross-sectional lung function data (Table 1) were included, as were 12 articles with longitudinal lung function data (Table 2).

### Air Pollution Exposure

Ambient (outdoor) air pollution constitutes a complex mixture of compounds, which vary in concentration depending on sources, geography, topography, wind direction and speed, temperature, ultraviolet radiation, and relative humidity. The concentrations of pollutants may be correlated both in time and space, because they come from the same sources and are distributed similarly. Therefore, in studies of health effects, it may be difficult to discern the importance of one pollutant from the other.

Ambient air pollution consists of organic and inorganic liquid and solid particles suspended in air (particulate matter—PM), as well as different type of gases such as ozone (O_3_), nitrogen oxides (NO_x_), and carbon monoxide (CO), as well as vapors, as volatile organic carbons (VOCs) [55].

Classification of particles is often according to size, and this provides information about possible health effects. For example, particulate matter with an aerodynamic diameter of less than 10 μm (PM_10_) is inhalable and reaches the lower airways and is subdivided into PM_coarse_ (PM with a diameter between 2.5 and 10 μm), which reach the proximal airways, and PM_2.5_ (PM < 2.5 μm), which reach the more peripheral regions of the lungs where gas exchange occurs. Ultrafine particles consist of those with a diameter of less than 0.1 μm, which contribute little to the total mass but are higher in numbers, have a large surface area, and are suggested to reach past the alveolar wall into the blood circulation [56].

### Proposed Mechanisms Related to Health Effects

The exact mechanisms by which air pollution affects the lungs and airways are not known. It has been hypothesized that oxidative stress and airway inflammation are important processes [57]. It has for example been suggested that inhaled particles provoke the generation of reactive oxygen species. This, as well as direct damage by highly oxidative gases such as ozone and NO_2_, induces oxidative stress and inflammatory responses [55]. Epigenetics has been proposed as one of the links between exposure to air pollution and respiratory health effects, for example through methylation of genes involved in immune-mediated inflammatory response [58, 59]. In studies of human histological lung tissue, correlations have been observed between exposure to high PM levels and small airway remodeling by greater amounts of fibrous tissue and smooth muscle cells [60].

Studies investigating exhaled nitric oxide in humans support the notion that inflammatory processes may play a role for the observed respiratory health effects by exposure to air pollution [61]. Nitric oxide is an established biomarker of airway inflammation, and several studies show a relation between exposure to air pollution and increased levels of the exhaled fraction of nitric oxide (FeNO). This has been observed for short-term exposures, long-term exposures, markers of traffic-related air pollution, and even in children with no history of airway damage [61].

### Air Pollution Exposure and Lung Function in Children and Adolescents

A number of epidemiological studies have investigated the association between long-term exposure to traffic air pollution and lung function in children and adolescents, and evidence on the negative effects of exposure to air pollution on respiratory health is accumulating. The studies with individual exposure assessment and in case of aggregated data use, studies with at least three communities included are summarized in Tables 1 (cross-sectional studies) and 2 (longitudinal studies).

**Table 1:** Cross-sectional studies of long-term exposure to air pollution and lung function (LF) in children and adolescents

Author, year	Country (cohort name)	N	Ages (years)	Exposure(s)	Outcome(s)	Subgroup(s)	Main findings
Barone-Adesi, 2015 [11]	UK (CHASE)	4884	9–10	PM_10_,PM_2.5_, NO_x_, NO_2_, NO,O_3_,O_x_ (annual average for current year based on dispersion modeling at home address), traffic-proximity metrics.	FEV_1_, FVC, FEF_25,50,75_	None	Negative non-significant association between all pollutants (except O_3_, traffic proximity) and FEV_1_ and FVC. Stronger effect for FVC.
Brunekreef, 1997 [12]	Netherlands	877	7–12	Distance to motorway, traffic density, school indoor measurements PM_10_, NO_2_	FEV_1_, FVC, PEF, FEF_25–75_	Gender	Negative association of truck traffic on LF, stronger effects in girls
Cakmak, 2016 [13]	Canada	1528	9–11	Traffic-counts and PM_2.5_, NO_2_, SO_2_ (LUR-annual averages)	FEV_1_, FVC, eNO	SES	Effects on FEV_1_ and FVC in low-income group
Dales, 2008 [14]	Canada	1613	11	NO_2_, PM_2.5_, PM_coarse_, PM_2.5 soot_, SO_2_ (LUR annual average at current address), roadway density	FEV_1_, FVC, eNO	Asthma	Significant positive effects for roadway density and PM on eNO, stronger in subjects with asthma
Dockery, 1989 [15]	USA (The 6 cities study)	5422	10–12	TSP, PM_1.5_, PM_2.5_,SO_4_, SO_2_, O_3_, and NO_2_ (central city monitoring stations, daily, monthly, annual mean)	FEV_1_, FVC, FEV_0.75_, and FEF_25–75_	Asthma and wheeze	Null effects for lung function
Eeftens, 2014 [16]	Europe (ESCAPE)	4659	6–8	PM_10_ and PM_2.5_: elemental composition (LUR at current addresses)	FEV_1_, FEV_0.5_, FVC, PEF	Asthma	Small effects related to nickel and sulfur. Heterogeneity across cohorts. Stronger effects in children with asthma
Eenhuizen, 2013 [17]	Netherlands (PIAMA)	880	4	NO_2_, Pm_2.5_, _soot_ (LUR annual averages at birth address)	Interrupter resistance (R_int_)	Gender, Parental allergy	Positive association between air pollution at birth address and R_int_, no effect modification
Frye, 2003 [18]	Germany	1911	11–12	Total suspended particles (TSP) and SO_2_—daily and annual averages—community monitoring	FEV_1_, FVC	Gender	Effects of reduction in TSP and an increase in FVC and possible to a smaller degree in FEV_1_, especially in girls
Fuertes, 2015 [19]	Germany (LISA and GINI)	2266	15	NO_2_, PM_2.5_, PM_2.5_absorbance, O_3_ (LUR - annual averages at birth, 10-year and current residential address)	FEV_1_, FVC, FEF_25_, FEF_50_, FEF_75_, FEF_25–75_, PEF, < LLN	Gender, Asthma	No convincing overall effects but indications of effects from current exposure in subjects with asthma
Gao, 2013 [20]	China	3168	8–10	PM_10_, SO_2_, NO_2_, O_3_ (lifetime and current annual averages from local monitoring stations)	FEV_1_, FVC, FEF_25–75_, FEF_75_	Gender	FEV_1_, FEF_25–75_, and FEF_75_ were significantly lower in boys in high-pollution district than in low-pollution district
Gehring, 2013 [21•]	Europe (ESCAPE)	5921	6–8	NO_2_, NO_x_, PM_10_, PM_2.5_, PM_coarse_, PM_2.5_ absorbance (LUR—annual averages at birth and current address)	FEV_1_, FEV_0.5_, FVC, PEF	Asthma, gender sensitization	Estimated levels of NO_2_, NO_x_, PM_2.5 absorbance_, and PM_2.5_ at the current address, but not at the birth address, were associated with small decreases in lung function.
Gehring, 2015 [22]	Netherlands (PIAMA)	3702	11–12	PM constituents, PM_2.5_, PM_10_ (LUR—annual averages at birth and current address)	FEV_1_, FVC, FEF_25–75_	Allergy, SES	Copper and iron (from PM_2.5_) at current address was negatively associated with FEV_1_, also FEF_25–75_ (with copper from PM_10_)
Hirsch, 1999 [23]	Germany	1137	9–11	SO_2_, NO_2_, CO, benzene, O_3_ (modeled previous year averages based on measurements stations—residential and school addresses)	FEV_1_, FEF_25–75_, BHR	None	Null effects for lung function and BHR
Hoek, 2012 [24]	Multicenter	22,809	6–12	PM_10_, NO_2_, and SO_2_ from local monitoring stations, approximately 1 year previous spirometry test	FEV_1_, FVC, FEF_25–75_, PEF	Gender, wheeze, sensitization	Null effects in combined analyses on lung function
Islam, 2011 [25]	USA (CHS)	1399	11	NO_x_, NO_2_, NO (LUR at school and resident)	FEV_1_, FVC	Parental stress level, no asthma	Sign negative association of NO_x_ and FEV_1_, in high-stress households. Effect remained in non-asthmatics
Janssen, 2003 [26]	Netherlands	1726	7–12	Truck/car traffic counts, PM_2.5_ and NO_2_ (estimated averages during previous year based on measurements at schools)	FEV_1_, FVC, FEF_25–75_, BHR	Sensitized, +BHR	Null effects for lung function and BHR
Lee, 2011 [27]	Taiwan (TCHS)	3957	12–13	CO, NO_x_, NO, NO_2_, O_3_, SO_2_, PM_2.5_, PM_10_ (community-based monitoring data—annual, and monthly averages)	FEV_1_, FVC, FEF_25–75_, PEF	Gender, asthma	Sign negative association of annual CO, NO_x_, NO_2_, and NO with FVC and FEV_1_, especially in boys
Morales, 2015 [28••]	Spain (INMA)	620	4.5	NO_2_ and benzene (LUR; Trimester specific, first year of life, previous year, current (1 week))	FVC, FEV_1_, PEF, FEF_25–75_, < 80% predicted	Gender asthma, SES, allergy	Strongest association on LF after exposure in second trimester of pregnancy, especially among allergic children and those of low SES (negative associations, but not significant for the other time periods)
Neophytou, 2016 [29]	USA and Puerto Rico (GALA II and SAGE II)	1968	8–21	NO_2_, SO_2_, O_3_, PM_2.5_, PM_10_ (calculated at residents from 4 monitoring stations. Monthly, annual, and lifetime averages)	FEV_1_, FVC FEF_25–75_	Global genetic ancestry	Lifetime average and first year of life PM_2.5_ was associated with reduced FEF_25–75_ and FEV_1_
Nicolai, 2003 [30]	Germany	904	9–11	Proximity to traffic and traffic counts, benzene, NO_2_, and soot (estimated residential averages during previous year based on measurements stations)	Spirometry	SHS	Null effects for lung function and BHR
Nordling, 2008 [31]	Sweden (BAMSE)	2599	4	NO_x_, PM_10_, SO_2_ (first year of life averages based on dispersion model at residential addresses)	PEF	Gender, wheezing	Effects on PEF, most strong for PM_10_. No effect modification
Oftedal, 2008 [32••]	Norway	2307	9–10	NO_2_, PM_10_, PM_2.5_ (early life, lifetime, and current annual averages based on dispersion model at residential addresses)	FEV_1_, FVC, FEF_25_, FEF_50_, PEF	Gender, asthma, ethnicity	Negative association between all time periods of exposures and PEF, FEF_25_, and FEF_50_, especially in girls. Slightly stronger effect from first year of life exposures. Stronger PM, and weaker NO_2_ effects in asthmatics
Peters, 1999 [33]	USA	3293	9–16	NO_2_, PM_10_,O_3_, acid vapor (12 communities—local monitoring stations during yrs. 1986–1990, and year 1994)	FEV_1_, FVC, FEF_25–75_, PEF	Gender	Effects on FEV_1_, FVC, and FEF_25–75_ in girls. Stronger effects from current exposure
Raizenne, 1996 [34]	USA and Canada	10,251	8–12	Particles and gaseous pollutants. Community level: previous year averages based on measurements	FEV_1_, FVC, FEV_0.75_, FEF_25–75_, PEF, FVC < 85% predicted	Gender	Effects on FEV_1_, FVC and FEF, especially strong in association with particle acidity. No gender difference.
Rice, 2016 [35]	USA (Project Viva)	614	8	Proximity to major road, PM_2.5_ (hybrid model) and BC (LUR):LUR and hybrid model: first year of life, life time, previous year	FEV_1_, FVC, bronchodilator response	Gender, SES, SHS, asthma	Prior year and lifetime PM and BC—significant with FVC (and none significant with FEV_1_), also higher OR of <80% predicted. No effect modification by asthma, gender or SHS. Stronger effects in high-income households
Schultz, 2012 [36]	Sweden (BAMSE)	1924	8	PM_10_, NO_x_ (first year of life, 1–4 years, and 4–8 years averages based on dispersion modeling (DM) at addresses)	FEV_1_, FEV_0.5_, FVC, < 80%, < 85% predicted	Gender, sensitization	Associations between first year of life exposure and mainly FEV_1_
Schultz, 2016 [37•]	Sweden (BAMSE)	2415	16	PM_10_, NO_x_ (first year of life, previous year averages based on DM at addresses)	Impulse oscillometry measurements	Gender, asthma, sensitization,	Associations between first year of life exposure and indices related to function in the small airways, especially in those with asthma
Schwartz, 1989 [38]	USA	3922	6–24	TSP, O_3_, NO_2_, SO_2_ (community-level annual averages)	FEV_1_, FVC, PEF	No	Effects on lung function from all pollutants (except SO_2_). Threshold effects for TSP and O_3_.
Sugiri, 2006 [39]	Germany	2574	5–7	TSP and SO_2_—daily and annual averages—community monitoring (background levels), and residential distance to major road	TLC, airway resistance	No	Better total lung capacity (TLC) when TSP decreased, but not related to distance from traffic
Svendsen, 2012 [40]	USA	1529	10	NO_2_- and diesel-related compounds (LUR at school and residential address)	FEV_1_, FVC, < 85% predicted	Gender, Asthma, SHS	Negative associations between NO_x_ and FVC. Increased OR of <85% predicted FEV_1_ and FVC. Stronger effect in children not exposed to SHS
Wang, 2015 [41]	The Netherlands (PIAMA)	1058	8	NO_2_, PM_2.5_, PM_10_, PM_2.5 soot_ (LUR and DM—birth and current address)	FEV_1_, FVC, PEF	No	Negative associations all pollutants and FEV_1_ and FVC, not PEF
Wjst, 1993 [42]	Germany	4320	9–11	Traffic-density in school district (as a proxy for long-term exposure—minimum of 5 years at current residence)	FEV_1_, PEF, FEF_25_, FEF_50_, FEF_75_,	No	Effects on PEF and FEF_25_, and FEF_50_.

**Table 2:** Longitudinal studies of long-term exposure to air pollution and lung function in children and adolescents

Author (year)	Country (cohort)	Number	Age at start (follow-up time)^a^	Exposure(s)	Outcome(s)	Subgroup(s)	Main findings
Avol, 2001 [43]	USA (CHS)	110	10 (5)	PM_10_, NO_2_, O_3_ (annual averages from community monitoring stations)	FEV_1_, FVC, FEF_25–75_, PEF	No	LF (not FVC) lowered when subjects moved to high PM_10_-level areas and increased when moving to low PM_10_ areas.
Gauderman, 2000 [44]	USA (CHS)	3035	Fourth, 7th and 10th graders (4)	PM_10_, PM_2.5_, PM_coarse_, NO_2_, O_3_, inorganic acid vapor (annual averages from community monitoring stations)	FEV_1_, FVC, FEF_25–75_, FEF_75_	Gender, Asthma	Most effects in fourth graders: deficits in LF growth related to increase in all pollutants (except O_3_). No effect modification by asthma or gender
Gauderman, 2002 [45]	USA (CHS)	1678	Fourth graders (4)	PM_10_, PM_2.5_, PM_coarse_, NO_2_, O_3_, inorganic acid vapor, elemental carbon (annual averages from community monitoring stations)	FEV_1_, FVC, FEF_25–75_, FEF_75_, PEF	Gender, Asthma	Replication but less strong effects of results from Gauderman 2000. Effects from O_3_. No effect modification by asthma or gender
Gauderman, 2004 [46••]	USA (CHS)	1759	10 (8)	PM_10_, PM_2.5_, PM_coarse_, NO_2_, O_3_, inorganic acid vapor, elemental carbon (annual averages from community monitoring stations)	FEV_1_, FVC, FEF_25–75_, FEF_75_, FEV_1_ < 80% predicted	No Asthma, No smoking	Effects of NO_2_, acid vapor, EC, and PM_2.5_ on LF. Increased OR of <80% predicted if high exposed. Effect remained in non-asthmatics and non-smokers
Gauderman, 2007 [47]	USA (CHS)	3677	10 (8)	Proximity to traffic, regional measurements	FEV_1_, FVC, FEF_25–75_, FEF_75_, FEV_1_ < 80% predicted	Gender, No asthma, No smoking	Effects on FEV_1_ and FEF_25–75_ growth independently for proximity to traffic and regional levels. Effects mainly in boys. Effect remained in non-asthmatics and non-smokers
Gauderman, 2015 [48••]	USA (CHS)	2120	11 (4)	PM_10_, PM_2.5_, PM_coarse_, NO_2_, O_3_, (annual averages from community monitoring stations)	FEV_1_, FVC, < 90%, <85%, and <80% predicted	Gender, Asthma status	Improvements in FEV_1_ and FVC growth related to declining levels of PM_10_, PM_2.5_, and NO_2_. Stronger effects in boys
He, 2010 [49]	China	1983	8–10 (0.5)	PM_10_, SO_2_, NO_2_ (lifetime and current annual averages from local monitoring stations)	FEV_1_, FVC, FEF_25_, FEF_25–75_, FEF_75_	Gender	Children living in high polluted district showed significant deficits in FEV_1_, FEF_25_, and FEF_25–75_ growth. No effect modification by gender
Horak, 2002 [50]	Austria	860	6 (3)	PM_10_, NO_2_, O_3_ (6-month averages from community monitoring stations in proximity to school)	FEV_1_, FEF_25–75_	Asthma, ETS	Effects of NO_2_ and O_3_ on FVC and FEV_1_. Summer PM_10_ was negatively associated with growth of FEV_1_ and FEF_25–75_. No clear effect modification
Mölter, 2013 [51•]	UK (MAAS)	1185	3 (8)	PM_10_ and NO_2_ (different time windows over the life course and previous year, individual estimates by micro-environmental model based on LUR)	sRAW, FEV_1_, bronchodilator	No	Small but significant deficits in growth of FEV_1_. Stronger effects after bronchodilator treatment, especially in relation to early life exposures
Neuberger, 2002 [52]	Austria	3451	Elementary school age (5)	NO_2_, SO_2_, TSP (calculated at school addresses from regional monitoring stations)	FEV_1_, FVC, FEF_25_, FEF_25–75_, FEF_75_	No	Faster FEF_25_, and FEF_25–75_ growth in areas with NO_2_ level reductions
Rojas-Martinez, 2007 [53]	Mexico	3170	8 (3)	PM_10_, NO_2_, SO_2_, O_3_ (6-month average from community monitoring stations in proximity to school)	FEV_1_, FVC, FEF_25–75_	Gender	Reduced FEV_1_ and FVC growth (also for ratio) in areas with high levels of PM_10_, NO_2_, and O_3_. No convincing gender difference.
Schultz, 2016 [54••]	Sweden (BAMSE)	2278	8 (8)	PM_10_, NO_x_ (first year of life, 1–8 years, 8–16 years of averages based on DM at addresses)	FEV_1_, FVC,< LLN	Gender, asthma, sensitization, ETS, maternal smoking during pregnancy	Associations between first year of life exposure and FEV_1_ at 8 and 16 years of age, but not with the change between 8 and 16 years. Effect modification by ETS and maternal smoking during pregnancy and/or infancy

^a^In years if not otherwise stated

Most studies report of lung function data from a single point in time (see Table 1; 32 cross-sectional studies vs. 12 with longitudinal data). The most commonly reported index is forced expiratory volume in 1 s (FEV_1_), representing mainly the mechanical properties of the large and medium-sized airways, followed by the forced vital capacity (FVC), reflecting lung size. Linear statistical models are generally used. Studies have compared lung function levels in children living in different communities with varying levels of ambient air pollution measured at central monitoring stations or at schools [15, 18, 27, 30, 33, 34, 38, 39, 43–45, 46••, 49, 50, 52, 53]. Traffic measurements such as traffic density or proximity to highways have also been used as exposure estimates [12, 14, 26, 30, 39, 42], as well as modeling individual data using land use regression (LUR) models [13, 14, 16, 17, 19, 22, 25, 28••, 35] or dispersion models (DMs) [31, 32••, 36, 37•, 41, 54••]. Most but not all studies [15, 23, 26, 30] have observed negative impact from traffic-related air pollution on lung function.

A general finding seems to be a larger effect estimate observed for FEV_1_ than for FVC. This pattern has been observed in studies from Austria [52], China [20], the USA [25, 44, 45, 47, 48••], Puerto Rico [29], Sweden [36, 54••], and Norway [32••], even though many of these studies observed the strongest effect estimate for mid-expiratory flows (FEF_25–75_). However, FVC has in some studies shown stronger associations than FEV_1_ [33, 35, 40, 41, 53].

The effect estimates across studies, i.e., change in mean values of lung function, are not always straightforward to compare and interpret, as the exposure usually differ between studies. For example, the mixture of components may differ between the studies due to differences in car fleet, road wear, use of studded tires, and fuel composition. Most epidemiological studies using modeled exposures cannot separate the different components of the emissions effectively [16, 22]. Because of the diversity across studies, we desist from reporting quantitative summary of effects. However, when reduction in lung function is reported in association with traffic-air pollution exposure, it is ususally with deficits of a few percent (0.5–3%) [10, 11, 21•].

An average reduction in lung function of a few percent has from an individual perspective likely only minor physiological effects. A small shift in the population distribution of lung function may, however, increase the prevalence of subjects presenting lung function values below clinical thresholds, as seen in studies that show increased risk of having less than 80 or 85% of the predicted FEV_1_ and/or FVC values [28••, 34–36, 40], or less than the lower limit of normal (<−1.645 SD) [54••]. A substantial improvement in public health may subsequently follow decreased air pollution levels, and this effect was recently seen in a publication from the Children’s Health Study in California. In this study, Gauderman and colleagues initiated and followed three cohorts during separate calendar periods, at the same time as air pollution levels improved [48••]. The authors observed that the risk of exhibiting FEV_1_ values below 80% of predicted at 15 years of age declined from 7.9 to 6.3 to 3.6% across the three time periods, indicating a substantial improvement in public health following decreased levels of air pollution.

### Timing of Air Pollution Exposure

Although several studies highlight the importance of exposures prenatally or during infancy for subsequent respiratory health [9, 62, 63], until recently, only a few studies had investigated exposure during the infancy period in relation to lung function in children [31, 32••] and none in adolescents. The majority of published cross-sectional and longitudinal studies have only investigated later life exposures (usually around school ages, see Tables 1 and 2), and the potential dynamic changes of exposure to air pollution during the life-course and subsequent influences on lung function growth remain largely unknown.

To date, there are eight studies that have reported on the relative impact of early life vs. current exposure to traffic-related air pollution. A Norwegian cohort study of 2307 9- and 10-year-olds showed that both early life and lifetime exposure to PM_10_ and NO_2_ were negatively associated with lung function [32••], even though early life exposure had slightly stronger effects. A meta-analysis of five European birth cohorts within the ESCAPE collaboration showed associations mainly with exposures at the current address and lung function at 6–8 years of age, while no significant association was seen with exposures at the address at birth [21•]. Mölter and colleagues have followed 1185 children from birth to 11 years of age within the MAAS cohort [51•] and observed strong effects mainly from exposure to PM_10_ during early life and post-bronchodilator FEV_1_ at 5 and 11 years of age. Fuertes et al. report from a German cohort of 2266 15-year-olds overall null findings irrespective of exposure time periods [19]. In a recent report, Rice and co-authors investigated 614 children (mean age 7.7 years) and concluded that the first year of life, lifetime, and past year exposure to PM_2.5_ and black carbon were all negatively associated with mainly FVC (not as strongly with FEV_1_), but that only the latter two time periods were statistically significant [35]. Studies from the Swedish birth cohort BAMSE report associations mainly from first year of life exposure to PM_10_ and/or NO_x_ with FEV_1_ at 8 years of age [36], at 16 years of age [54••], and with impulse oscillometry measurements at 16 years of age [37•].

In summary, the evidence of a particular important air pollution exposure time window for subsequent lung function appears inconclusive, and findings from studies published to date rather support that exposure over the entire age range is of importance. Most longitudinal studies are supporting this observation, with attenuated lung function growth in relation to air pollution exposure during later childhood and adolescence [46••, 51•, 53] being reported, as well as recovery of previous deleterious effects in subjects moving to less polluted areas [43]. In one study (from the BAMSE cohort), no association was observed for the change of lung function between childhood and adolescence, for any of the explored life-time exposure time windows [54••]. The authors highlight the fact that the levels of air pollution decreased during the course of the study, limiting the possibility of discerning the importance of exposure during adolescence. Most studies on lung function growth focus only on exposure during later life [43–45, 46••, 49, 50, 53], and the potential impact of exposures during the first year of life in these cohorts is therefore unknown.

### Small Airways

The small airways are by convention defined as those with an airway diameter of 2 mm or less (in adults). This corresponds to approximately airway generation 8 and more distally and comprises the more peripheral conducting airways (up to generation 15), as well as the bronchioles and alveolar regions where gas exchange occurs. For many lung diseases, like chronic obstructive pulmonary disease (COPD) and asthma, the small airways are a major site of pathology [64, 65•], and the severity of disease is often rather significant before changes in spirometry measurements appear [66]. As the small airways are relatively difficult to study, they are sometimes referred to as “the quiet zone” of the lung [67].

Most investigations linking air pollution exposure to lung function have employed measurements of total airway resistance and large rather than small airway function measured using spirometry [68]. In experimental studies on mice, small aerosol particles of a size range typical of traffic-related air pollution are deposited in the small airways [69].

There are reports of associations between exposures to air pollution and forced mid-expiratory flows, like FEF_25–75_, suggesting that the results represent small airway effects [22, 44, 49, 50, 52]. Although flows measured in the middle or at the end of a forced expiration generally offer insight of the more peripheral airway, the interpretation has lately been subject to debate [70]. Abnormalities in these flow indices are not specific to small airway disease [71], and it has been suggested that the mid-expiratory flows do not contribute additional information over what is provided by FEV_1_ and FVC [68].

An alternative measure of the small airways that may be feasible in an epidemiological setting is assessment of the residual volume (RV) using plethysmography. This index provides sensitive measure of gas trapping and hyperinflation, although not specific to small airway involvement [67]. Inert gas washout and forced oscillation techniques, such as impulse oscillometry (IOS), are methods that may discriminate between large and small airway effects [67, 72, 73]. In recent years, these techniques have become commercially available and easily accessible.

Despite advances with peripheral airway assessment, it is rarely investigated whether exposure to traffic-related air pollution influences small airway function. In a panel study of 163 children aged 7–10 years in Austria, IOS indices related to peripheral airway resistance was increased in relation to short-term exposure to air pollution [74]. Following 174 adults exposed to dust/fume from the World Trade Center collapse with airway symptoms and normal spirometry revealed abnormalities in the peripheral airways assessed with IOS in 68% of the subjects [75]. In the same group of individuals, RV/total lung capacity was elevated in approximately 33% of subjects, also indicative of small airway involvement. In 2415 children from the Swedish BAMSE cohort, NO_x_-exposure from the first year of life was associated with increased resistance in indices related to peripheral airway function also assessed with IOS at 16 years of age [37•].

To summarize, few studies have addressed small airway disease in relation to air pollution exposure to date, although associations between exposure and small airway involvement have been reported. Given the importance of small airway function in asthma and COPD, further studies are warranted.

### Susceptible Subgroups

In our modern society, everybody is more or less exposed to air pollution. However, certain people or subgroups in the population may be more susceptible to negative outcomes following exposure. For the studies included in the present review, we have focused on sub-analyses with respect to asthma, sensitization, and gender status.

#### Gender Differences

Gender differences on lung function in response to air pollution or other environmental stimuli may be present due to inherited differences in lung and airway development. For example, surfactant production starts earlier in the lungs of neonatal females than males [76], which may be one reason why infant females have lower airway resistance and higher air-flow rates compared to infant males [77]. In addition, males present already at birth with a higher total number of alveoli and alveolar surface area than females do, but at the same time, the growth of the airways lags behind, resulting in relatively more narrow airways in infant to adolescent males compared with females (known as dysanaptic growth) [76]. Males may, therefore, during infancy, childhood, and early adolescence have a pulmonary phenotype more susceptible to the deleterious effects of air pollution exposure [78]. During puberty, however, the risk in males compared with females may become reversed as several studies indicate influence by estrogens (female sex hormone) on increased incidence and severity of asthma [79]. Thus, a gender-related vulnerability to the detrimental effects from exposure to air pollution may be influenced by age.

Several of the published epidemiological studies within the area of this review have presented stratified analyses based on gender, but results have not been consistent. Six studies show stronger effect of air pollution exposure in males compared with females [20, 27, 37•, 47, 48••, 54••]. There are almost as many reporting stronger effects in girls [12, 18, 32••, 33] and a majority showing no differences in associations [17, 34, 40, 44, 45, 49, 53]. In addition, the mixed results regarding the role that gender has in relation to air pollution—lung function association is not obviously explained by age, as the observed differences in results cover the entire age range. Based on the published studies to date, there seems to be no conclusive evidence regarding gender-pollution interaction effects on lung function.

#### Asthma

Data regarding asthma as an effect modifier for the association between traffic air pollution and lung function are accumulating, although still limited. The majority of reports from the Children Health Study in the USA show no effect modification by asthma [25, 44, 45, 46••, 47, 48••]. Additional studies also show no differences in relation to asthma status: Svendsen and colleagues including 1529 10-year-olds in El Paso, Texas [40]; Schultz and colleagues from the 16-year BAMSE cohort [54••]; and Rice and colleagues in 614 children aged 8 years [35]. Stronger effects in those with asthma are, however, suggested from several studies [14, 19, 32••, 37•]. For example, Eeftens report from the ESCAPE collaboration small effects from nickel and sulfur (part of the air pollution mixture) on lung function especially in children with asthma [16]. Dales et al. observed stronger associations between roadway density and particles on the exhaled nitrogen oxide in children with asthma, indicative of increased airway inflammation [14]. Stronger association for children with asthma was also seen in the BAMSE cohort for small airway indices assessed with IOS [37•] (but not for FEV_1_ [54••]).

There is a risk that many studies are underpowered to detect significant effect modification by asthma status, which may explain some of the null findings. Weaker effects in children with asthma is, to our knowledge, seldom reported [32••], which may support the conclusion that if effect modification by asthma exists, it points towards an increased effect in children with asthma.

#### Sensitization

Effect modification of air pollution-lung function associations by sensitization has not been well investigated in epidemiological studies. In one study, Morales and colleagues found stronger associations on FEV_1_ in allergic (allergic asthma, atopic dermatitis, eczema, or allergic rhinitis) 4.5-year-olds exposed to air pollutants during pregnancy and lifetime, compared with non-allergic children [28••]. Janssen and colleagues reported that associations between truck-traffic counts and respiratory symptoms were only observed among children with bronchial hyper-responsiveness and/or sensitization [26], indicating a sensitive subgroup. However, no associations were observed in the same study regarding spirometry outcomes. In studies from Schultz et al. from the BAMSE cohort, the results from subgroup analyses differed depending on whether the outcome was assessed at 8 or 16 years of age. A stronger association between the first year of life exposure to PM_10_ and FEV_1_ at 8 years of age was suggested in those sensitized than not sensitized. However, the effect modification was not statistically significant [36]. At 16 years of age, no association difference between sensitized vs non-sensitized subjects was seen [54••]. As part of the ESCAPE project, stratified analyses based on sensitization status for the relation between air pollution exposure and lung function at 6–8 years of age did not reveal evidence of any effect modification [21•].

Evidence supporting sensitization as an effect modifier comes from a high-risk cohort study, where the authors observed that early exposure to allergens in combination with secondhand smoke exposure increased the risk for incident asthma, compared with having neither of the exposures [80]. Such findings are supported by controlled exposure studies [81, 82•].

The mechanisms behind a potential effect modification are not clear, but it has been suggested that genetically susceptible children more often present with impaired epithelial barrier function, which subsequently increases the airway’s vulnerability to early life air pollution exposure [83]. Epigenetic effects on DNA have been proposed as a potential link by which sensitization-pollution interaction influences the lung function [84]. Based on the identified epidemiological studies included in this review, evidence for effect modification by sensitization status of the relation between air pollution exposure and lung function remains inconclusive.

## Conclusions and Future Directions

**Fig. 1:**
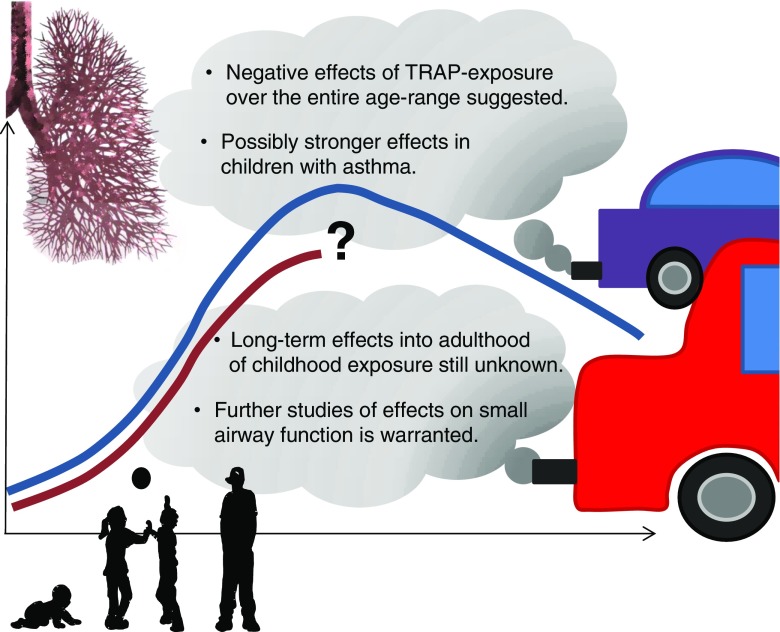
A schematic illustration of effects on lung function from long-term traffic-related air pollution (TRAP) exposure. The *Y-axis* corresponds to lung function and the *X-axis* corresponds to time (age). The *blue line* illustrates normal lung function growth and decline, with a maximum in young adulthood. The *dark red line* illustrates lung function growth slightly less than normal due to exposure of traffic-air pollution. The long-term effect of air pollution exposure during childhood remains largely unknown

As much as 85% of the world’s population live in cities with outdoor air pollution levels exceeding the WHO Air Quality Guidelines for PM_10_ [2]. The trend towards global urbanization implies that more and more people will become highly exposed if further control of emissions is not applied. We conclude that early life and school-age exposure to air pollution has a negative impact on lung function, at least up to adolescence (Fig. 1). Whether these lung function deficits persist into adulthood, and subsequently result in a reduced maximally attained lung function, or a shortened growth phase, remains unknown. However, based on prior studies of lung function changes over time, these studies suggest that these affected children will continue to have lower lung function than those who were exposed to lower levels of pollutants, especially if they continue to be exposed. There is therefore a need to follow pregnancy and birth cohorts with air pollution data up to adulthood when the plateau of lung function is reached. The relative impact of pregnancy vs. early life exposure is sparsely investigated and when initiating new cohorts, efforts should be made to capture both pregnancy and lifetime exposures and measure lung function already in infancy. Findings from studies published to date support that exposure over the entire childhood age range seems to be of importance for lung function development. In this review, we have also evaluated potential effect modification by gender, asthma status, and sensitization but could not find any conclusive data to support evidence of specific sup-group effects. Most studies have used spirometry indices to evaluate lung function in relation to air pollution exposure. A handful of recent studies have used other methods such as impulse oscillometry techniques to also assess small airway involvement, and further research in this area is warranted.
